# *In vivo* Mammalian Alkaline Comet Assay: Method Adapted for Genotoxicity Assessment of Nanomaterials

**DOI:** 10.3389/ftox.2022.903896

**Published:** 2022-05-30

**Authors:** Renato Cardoso, Maria Dusinska, Andrew Collins, Mugimane Manjanatha, Stefan Pfuhler, Marilyn Registre, Rosalie Elespuru

**Affiliations:** ^1^ MilliporeSigma, Rockville, MD, United States; ^2^ Health Effects Laboratory, Department of Environmental Chemistry, Norwegian Institute for Air Research, Kjelle, Norway; ^3^ Department of Nutrition Institute of Basic Medical Sciences, University of Oslo, Oslo, Norway; ^4^ Division of Genetic and Molecular Toxicology Food and Drug Administration, National Center for Toxicological Research, Jefferson, AR, United States; ^5^ Global Product Stewardship, Human Safety Procter and Gamble Mason Business Centre, Mason, OH, United States; ^6^ Charles River Laboratories, Senneville, QC, Canada; ^7^ Division of Biology, Office of Science and Engineering Laboratories, Center for Devices and Radiological Health, Chemistry and Materials Science, U.S. Food and Drug Administration, Silver Spring, MD, United States

**Keywords:** nanomaterial, comet assay, single cell gel assay, genotoxcicity, DNA damage, hazard identification

## Abstract

The *in vivo* Comet assay measures the generation of DNA strand breaks under conditions in which the DNA will unwind and migrate to the anode in an electrophoresis assay, producing comet-like figures. Measurements are on single cells, which allows the sampling of a diversity of cells and tissues for DNA damaging effects. The Comet assay is the most common *in vivo* method for genotoxicity assessment of nanomaterials (NM). The Method outlined here includes a recommended step-by-step approach, consistent with OECD 489, taking into consideration the issues impacting assessment of NM, including choice of cells or systems, handling of NM test articles, dose determination, assay methods and data assessment. This method is designed to be used along with the accompanying “Common Considerations” paper, which discusses issues common to any genotoxicity assay using NM as a test article.

## Introduction

The methods found in this issue of *Frontiers in Toxicology* are devoted to Nanomaterials (NM) assessment. Four papers in the series are a follow-up to the analysis and critique of the literature on genotoxicity assessment of NMs by an international group working together via the GTTC (Genetic Toxicology Testing Committees) of the Health and Environmental Sciences Institute (HESI) (Elespuru et al., 2018). Besides this method for *in vivo* assessment of genotoxicity, a paper describing “Common Considerations” as well as two other methods for *in vitro* mammalian mutagenicity or clastogenicity are described in separate papers.

Although *in vitro* genotoxicity assays may be sufficient for assessment of genotoxicity in many contexts, *in vivo* assays may be uniquely valuable in assessing distribution or sequestration of NM, because of physical characteristics, that would not be detected otherwise. As noted in Elespuru et al. (2018), *in vivo* assays may be recommended if other data or circumstances indicate a NM distribution consistent with a sequestration or specific targeting. *In vivo* assays are not recommended as a primary screen for NM effects and should be justified. To fulfil reduction, refinement, and replacement (3 R’s) animal welfare requirements, this protocol can be integrated with other toxicological endpoints in a single animal study.

The *in vivo* alkaline comet (single cell gel electrophoresis) assay (hereafter called comet assay) is used to identify substances that cause DNA interactions that are generally related to DNA strand breakage. Cells or nuclei isolated from tissues of animals that have been exposed to the test article are embedded in agarose and lysed to form nucleoids. Electrophoresis causes DNA with breaks to extend towards the anode, giving the appearance of a comet under fluorescence microscopy; the relative intensity of DNA in the comet tail reflects the break frequency. The analysis does not allow for the discrimination of the origin of the strand break (e.g., direct break, intermediary DNA break introduced by repair mechanism, or indirect break resulting from inhibition of other bioprocess), or for the detection of DNA cross-links. See OECD Test Guideline (TG) 489 (OECD, 2016; Brunborg and Collins, 2020) for additional information.

For mechanistic studies, an additional step can be added to the standard comet assay - incubation with a DNA repair enzyme [e.g., 8-Oxoguanine glycosylase (OGG1) or formamidopyrimidine-DNA glycosylase (Fpg)] to detect oxidized DNA bases (Collins et al., 2017) [It is noted that the modified comet assay to detect DNA oxidation damage is not yet validated and the current OECD TG 489 (OECD, 2016) for the standard comet assay does not provide recommendations for the modified comet assay].

Although an *in vitro* comet assay could be informative, a universally accepted protocol or an OECD guideline for an *in vitro* version of the comet assay does not currently exist. For this reason, the *in vitro* comet assay is not recommended and not considered in this series of papers by the GTTC group.

## Test System

### Animal Strains

Various common laboratory strains of rodents (e.g., Sprague Dawley, Wistar Han, or F344 rats; CD1, BALB/c, or ICR mice) can be used for this assay. Animals should be 6–10 weeks old at the start of the treatment and within normal weight for their age (animal variation should not exceed 20% of the mean weight of each sex).

### Animal Housing and Feeding

The animals should have a minimally invasive unique identifier and be randomly assigned to treatment groups. Animals should be socially housed (up to four same sex animals per cage except if aggressive behavior is noted) in solid floor cages with hardwood chip bedding and micro-isolator bonnets. Animals should also be provided with items such as a hiding device and a chewing object. Conventional laboratory diets along with drinking water can be given *ad libitum* throughout the course of the study. The environment of the animal rooms is set to maintain a 12 h light cycle, temperature of 22 ± 3°C, relative humidity of 30–70%, and air changes of 10–15/h. The rats are provided standard pelleted food and purified water (e.g., distilled/deionized water) ad libitum (OECD, 2016). The care of animals and all animal experimental procedures will be performed in accordance with a study protocol approved by the Institutional Animal Care and Use Committee.

### Preparation of NM for Testing

NM characterization is generally required for data interpretation and publication. The NM should be prepared for testing, e.g., by sonication of particles and suspended in a non-toxic vehicle compatible with the test system (e.g., water for injection, physiological saline, ethanol, methylcellulose solution [See Elespuru et al., 2022]).

Since fluorescent lighting can induce oxidative damage, all procedures for the assay should be performed to protect the test articles from light exposure. If other than well-known vehicles for administration of NM are used, reference data demonstrating their compatibility with the test system should be provided. In the absence of previous data demonstrating no effect on comet induction, an initial study should be performed to qualify the vehicle.

## Preliminary Considerations

### Route of Administration

The route of administration may cover the intended or reasonably expected route of exposure to humans, if feasible. If more than one route of exposure is expected, then a rationale should be presented to select the route leading to higher exposure or that is expected to be the most sensitive. The gastrointestinal tract contains a layer of mucous which functions to prevent particles from contacting Payer’s patches and other entry portals. Gavage volumes should be minimized to avoid using large volumes of liquid vehicles which may “wash” away this protective coating.

### Proof of NP or NM Exposure and Cellular Uptake

If ADME (absorption, distribution, metabolism, elimination) studies on particle distribution are not undertaken, uptake of the NP into the cells analyzed should be assessed and the location of particles within the cells (i.e., nucleus or cytoplasm) determined if feasible. See the Common Considerations paper for additional information on uptake of NMs and methods for determination of exposure. In some cases, comet assay results may be positive in the absence of cellular uptake. This could reflect the consequence of artifacts such as tissue inflammation-related reactive oxygen species, toxicologically valid events such as breakdown of the material in the test environment, or the release of diffusible substances.

Proof of cellular uptake or target organ exposure is recommended for hazard identification. In cases where experimental data demonstrate that cellular uptake does not occur under the condition of testing, a negative test result may be consistent with a lack of exposure. A demonstration of exposure, lack of exposure, or systemic distribution is recommended for an evaluation of negative results.

### Dose-Range Determination

If a preliminary range-finding study is performed to support dose selection, it should be performed under similar conditions to those intended for the main study with the same species, strain, sex, test article preparation, route of administration, and treatment regimen. The study should aim to identify the maximum tolerated dose (MTD) without evidence of study-limiting toxicity for the duration of the study period (e.g., no death or evidence of pain, suffering or distress of the animals; no suppression of body weight gain, hematopoietic system toxicity, or increased inflammatory biomarkers). Inflammation is an important confounding factor for the comet assay. Thus, it is advisable to consider the impact of dose regimens that could lead to high levels of inflammation in the target tissue. Animals should be observed hourly for the first 4 h following each administration of test article. If the test article does not elicit toxicity under the conditions planned for the main experiment, the highest dose may be based on evidence of viscosity, NM dispersibility, aggregation or agglomeration. The study design and options for selection of the maximum dose can be found in the literature (Delmaar et al., 2015; Faria et al., 2018).

## Experimental Method [Refer Also to OECD 489, 2016]

### Controls

The comet assay is conducted with groups including vehicle control, at least three dose levels of test article, and a positive control. The selection of a positive control for the study should be based on demonstrating clear positive comet responses in the tissues of interest. Methyl methanesulfonate (CAS RN 66-27-3) is a widely used positive control as it has produced DNA strand breaks in all rodent tissues that have been studied. Other positive controls include ethyl nitrosourea (CAS RN 759-73-9), ethyl methanesulfonate (CAS RN 62-50-0), N-methyl-N′-nitro-N-nitrosoguanidine (CAS RN 70-25-7), 1,2-dimethylhydrazine 2HCl (CAS RN 306-37-6) and methylnitrosurea (CAS RN 684-93-5). Samples of positive target tissues for the respective positive controls can be found in the OECD TG 489. Positive controls other than these should only be selected if scientifically justified. Currently there is no NM specific positive control that has its response clearly defined. It is not necessary to administer concurrent positive control substances by the same route as the test article.

Each experimental group should have at least five animals (per sex, if both sexes are used) with no fewer than three animals acceptable for the positive control.

The route of administration to simulate human exposure may be selected from among the following options: dietary, drinking water, topical, subcutaneous, intravenous, oral (by gavage), inhalation, or implantation. Intraperitoneal injections of test article should be avoided unless specifically justified. The size of test animals should be considered when determining the maximum volume of liquid that can be administered in accordance with animal welfare legislation.

### Treatment Schedule and Dosing

Animals should be treated daily over a duration of two or more days and samples should be collected at 2–6 h after the last treatment, or at the time of maximum plasma concentration (if known). A longer treatment schedule may be used to allow the incorporation of the comet assay endpoint into other toxicology repeated dose assays, but one additional dose may need to be considered to satisfy the requirement for the collection of samples at 2–6 h after the last treatment.

### Observations

Clinical observations about the health of the animals need to be recorded at least once a day, considering the peak period of anticipated effects after dosing. Animals should be observed at least twice a day and at the end of the exposure period for morbidity and mortality. For longer duration studies, animals should be weighed at least once a week. If the test article is administered via feeding and drinking, then water and food consumption should be recorded. Animals showing signs of excessive toxicity should be euthanized prior to completion of the study and not used for comet analysis.

### Tissue Collection

Tissues considered for study should include the site of contact such as stomach/duodenum/jejunum for oral exposures, an organ representing systemic distribution such as liver, and an organ such as kidney where bioaccumulation may occur.

Animals should be sacrificed by procedures accepted by effective animal welfare regulation at the appropriate time after the last treatment. The selected tissue should be dissected, with resulting pieces used for comet preparation and for potential histopathology examination. Tissue for comet evaluation should be rinsed with 0.9% sterile saline, exsanguinated, and placed in rinsing buffer (see solution recipes at the end), and kept ice-cold until processed.

### Specimen Preparation

For all animals, soft tissues (e.g., liver, lung) are minced in cold homogenizing buffer (see recipes) to create a single cell suspension. Blood and bone marrow can be applied to the slide directly. For hard tissues (e.g., glandular stomach, duodenum), the epithelial cells are gently scraped into cold homogenizing buffer prior to passing the released cells through filtration, such as a sieve or mesh, to create a single cell suspension. Since UVA radiation from fluorescent lighting can induce oxidative damage, samples should be harvested in an environment protected from light. The samples are stored ice-cold until slide preparation.

### Preparation of Slides

The cell suspension is mixed with 1% low melting point agarose in phosphate-buffered saline (PBS) at 37°C to a final concentration of approximately 0.8%, and drops are placed on the coated slide. There are different formats ranging from 2–20 gel drops per slide. At least three gels per tissue per animal are prepared. Preferably, gels from each sample are set on different slides. Once gels are solidified, the slides are immersed in complete lysis solution in a light-proof box and placed at 4°C, until the electrophoresis step. Slides should be immersed in lysis solution for at least 1 h but can be stored in this solution at 4°C for up to 3 days [Formats allowing a higher throughput such as CometChip assay have been devised; they are not covered in this protocol.]

### Slide Analysis

**FIGURE 1:**
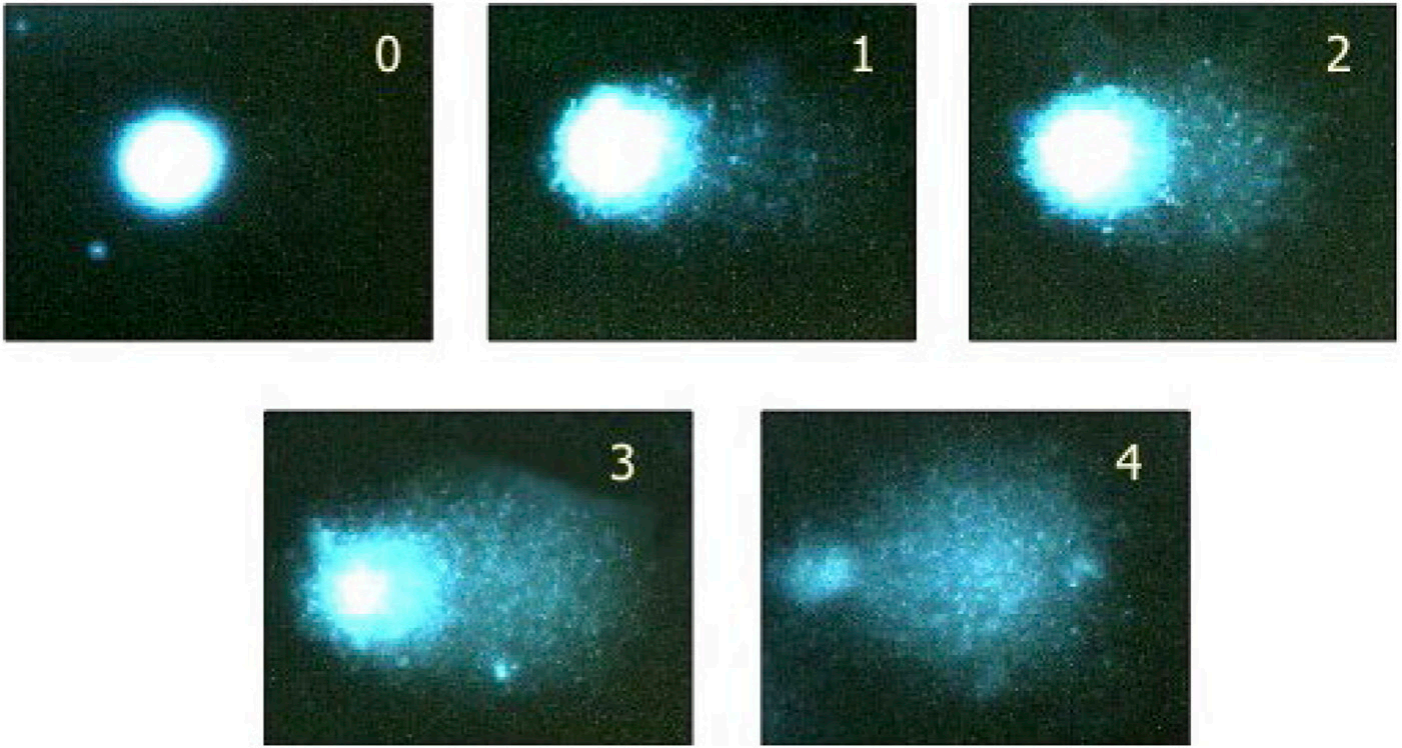
Visual scoring - an acceptable option if image analysis is not available. By eye, it is possible to classify comets into five categories, corresponding to these typical images. Examining 100 comets in this way, and giving scores corresponding to the classes (0, 1, 2, 3, 4), the total score will be between 0 and 400, roughly equivalent to 0–100% tail DNA. See Collins, 2004 (Collins, 2004) for more details, including a direct comparison of visual scoring with computer image analysis (Note: a class 4 comet would commonly be referred to as a ‘hedgehog comet’.) While visual scoring is possible, automated scoring is recommended, to avoid bias in data interpretation. Reprinted from Collins, 2004, by permission of Springer Nature.

Comets are scored quantitatively by an automated or semi-automated image-analysis system (e.g., Comet Assay IV) or by visual scoring (Figure 1). The slides are encoded to minimize potential operator bias. The slides are stained with an appropriate fluorescent stain (e.g., Propidium iodide, SYBR Gold, Green I.) and the comets (nucleoids) visualized using a fluorescence microscope at ×200 magnification.

Initially, gels are examined visually for any evidence of overt toxicity, e.g., an increase in background debris and/or an increase in the incidence of excessively damaged nucleoids (e.g., ‘hedgehogs’). These nucleoids cannot be quantified by image analysis, but their frequency should be recorded, along with nucleoids that have unusual staining artifacts or comets with non-spherical heads. Where practical, at least 150 nucleoids (excluding hedgehogs) should be analyzed per tissue per animal (e.g., 50 nucleoids on each of three gels). Several representative areas of the slide should be chosen to avoid bias; however, scoring at the edge of the slide should be avoided. Nucleoids should be scored for % DNA in tail (aka % tail intensity) (Burlinson et al., 2007; OECD, 2016).

### Expression of Results and Statistical Anaysis

According to consensus, the % tail intensity values should be presented as:

• The median % tail DNA for each slide and average for each animal.
• The mean of these median % tail DNA values and standard deviation of each group

The numerical data corresponding to % tail DNA are statistically evaluated in a tiered approach using two datasets. The first dataset includes the negative control group and the positive control group, to determine the validity of the assay. The second dataset includes the negative control group and the test article groups to determine the genotoxicity of the test article.

Detailed statistical approaches can be found in, (Lovell and Omori, 2008; Bright et al., 2011). Evaluation and interpretation of the results are described in the Common Considerations paper.

### Solutions

• Rinsing buffer for tissues: Hanks Balanced Salt Solution, containing 20 mM EDTA and 10% v/v dimethyl sulfoxide, pH 7.4,
• Homogenizing buffer for tissues: Hanks Balanced Salt Solution, with calcium, with magnesium, without phenol red, containing 20 mM EDTA and 10% v/v dimethyl sulfoxide, pH 7.4.
• Lysis solution: 2.5 M NaCl, 0.1 M EDTA, 10 mM Tris, pH 10 (with NaOH); 1% Triton X-100 added just before use.
• Electrophoresis solution: 0.3 M NaOH, 1 mM EDTA, pH > 13.

## Data Availability

The original contributions presented in the study are included in the article/Supplementary Material, further inquiries can be directed to the first author.

## References

[B1] Bright J. Aylott M. Bate S. Geys H. Jarvis P. Saul J. (2011). Recommendations on the Statistical Analysis of the Comet Assay. Pharm. Stat. 10, 485–493. 10.1002/pst.530 22127874

[B2] Brunborg G. Collins A. (2020). Guidance for Publishing Comet Assay Results. Mutat. Res. Genet. Toxicol. Environ. Mutagen. 854-855, 503146. 10.1016/j.mrgentox.2020.503146 32660819

[B3] Burlinson B. Tice R. R. Speit G. Agurell E. Brendler-Schwaab S. Y. Collins A. R. (2007). Fourth International Workgroup on Genotoxicity Testing: Results of the *In Vivo* Comet Assay Workgroup. Mutat. Research/Genetic Toxicol. Environ. Mutagen. 627, 31–35. 10.1016/j.mrgentox.2006.08.011 17118697

[B4] Collins A. El Yamani N. Dusinska M. (2017). Sensitive Detection of DNA Oxidation Damage Induced by Nanomaterials. Free Radic. Biol. Med. 107, 69–76. 10.1016/j.freeradbiomed.2017.02.001 28161308

[B5] Collins A. R. (2004). The Comet Assay for DNA Damage and Repair: Principles, Applications, and Limitations. Mol. Biotechnol. 26, 249–261. 10.1385/MB:26:3:249 15004294

[B6] Delmaar C. J. E. Peijnenburg W. J. G. M. Oomen A. G. Chen J. de Jong W. H. Sips A. J. A. M. (2015). A Practical Approach to Determine Dose Metrics for Nanomaterials. Environ. Toxicol. Chem. 34, 1015–1022. 10.1002/etc.2878 25565198

[B7] Elespuru R. Pfuhler S. Aardema M. J. Chen T. Doak S. H. Doherty A. (2018). Genotoxicity Assessment of Nanomaterials: Recommendations on Best Practices, Assays, and Methods. Toxicol. Sci. 164, 391–416. 10.1093/toxsci/kfy100 29701824

[B11] Elespuru R. K. Doak S. K. Collins A. R. Dusinska M. Pfuhler S. Manjanatha M. G. (2022). Common Considerations for Genotixicity Assessments of Nanomaterials. Methods, Frontiers in Toxicology Nanotoxicity. 10.3389/ftox.2022.859122 PMC9171035 35686044

[B8] Faria M. Björnmalm M. Thurecht K. J. Kent S. J. Parton R. G. Kavallaris M. (2018). Minimum Information Reporting in Bio-Nano Experimental Literature. Nat. Nanotech 13, 777–785. 10.1038/s41565-018-0246-4 PMC6150419 30190620

[B9] Lovell D. P. Omori T. (2008). Statistical Issues in the Use of the Comet Assay. Mutagenesis 23, 171–182. 10.1093/mutage/gen015 18385511

[B10] OECD (2016). Test No. 489: *In Vivo* Mammalian Alkaline Comet Assay. Paris: OECD Publishing.

